# The Effects of Social Networks of the Older Adults with Limited Instrumental Activities of Daily Living on Unmet Medical Needs

**DOI:** 10.3390/ijerph18010027

**Published:** 2020-12-23

**Authors:** Hye-Young Jang, Young Ko, Song-Yi Han

**Affiliations:** 1 College of Nursing, Hanyang University, Seoul 04763, Korea; white0108@hanyang.ac.kr; 2 College of Nursing, Gachon University, Incheon 21936, Korea; moodory@gmail.com; 3 Department of Nursing Science, Sunmoon University, Asan-si 31460, Korea

**Keywords:** older adults, social networks, instrumental activities of daily living, unmet medical needs

## Abstract

This study was conducted to identify the effects of social networks on unmet medical needs among older adults with limited instrumental activities of daily living (IADL) who live in a community. This study analyzed data from 2281 older adults with limited IADL from the 2017 National Survey of Older Koreans. Data were analyzed using descriptive statistics, X2 tests, *t*-tests, and logistic regression analysis. About 73.0% of the subjects were female and 15.8% of the subjects had experienced unmet medical needs. The predictors of unmet medical needs according to gender are as follows: annual household income, participation in social activities, and physical support for male subjects and annual household income, number of chronic diseases, living alone in a household, living with others in a household, frequency of contacting close friends, and emotional support for female subjects. The findings of this study will be utilized as a basis for establishing relevant measures to enable older adults to receive proper medical services by heightening the understanding of the gap between medical service use and the medical needs of older adults with limited IADL.

## 1. Introduction

The term “unmet medical needs” refers to a condition in which medical services perceived by the subject or determined by medical professionals as necessary have been insufficiently provided [1]. From the perspective that health is a universal right, unmet medical needs hold significance both individually and at the national level. National governments execute various policies and systems to provide opportunities for citizens to receive healthcare services when needed as a measure to guarantee that health is a universal right. Accordingly, unmet medical needs have been considered a key indicator in evaluating the performance of a healthcare system [2].

After the introduction of a national health insurance in 1989, an array of political implementations have been attempted to improve the accessibility and equity of the healthcare system in South Korea; however, the proportion of individuals experiencing unmet medical needs is still reported to be 8–17% [3], which indicates the ongoing problem of imbalance in unmet medical needs [4]. In particular, unmet medical needs are reported to be more prominent in socially or economically vulnerable groups [5], and therefore, measures to reduce the unmet medical needs of vulnerable populations require more attention.

The older adult population needs more medical services compared to other adult populations due to functional decline from aging and chronic diseases. It is also highly vulnerable to unmet medical needs due to a reduced income as a result of a reduction in economic activities. Unmet medical needs among the older adult population may lead to the occurrence of complications and disease deterioration, thus lowering quality of life [6] and leading to an increase in the risk of death [7,8]. Therefore, measures need to be sought to reduce unmet medical needs in the older adult population in order to improve their quality of life.

The activities of daily living (ADL) and the instrumental activities of daily living (IADL) refer to the basic functions needed for older adults to maintain an independent life [9]. In particular, IADL assesses abilities requiring a higher level of control such as using a phone, medicine administration, and using public transportation, which all are needed to carry out an independent life in a local community and are considered key indicators of the ability to maintain an independent life for older adults [9,10]. The deterioration of IADL leads to physical and spatial constraints in terms of movement [11], which restricts the activities needed to maintain social relationships, thus reducing interpersonal relationships and causing depression [12]. The deterioration of IADL further affects unmet medical needs [8] and lowers the quality of life for older adults [13]. Therefore, the unmet medical needs of older adults with restricted IADL should be examined, which will provide basic data for developing interventions that could enable older adults to receive appropriate medical services.

A social network refers to the connections between individuals who come into contact and interact with each other [14], and it plays a crucial role in the health of vulnerable groups, including low-income and disabled populations. According to a previous study [15], vulnerable groups exhibited different levels of health conditions depending on the levels of their social networks. This finding implies that the level of a social network may affect the use of medical services. However, there is insufficient research on the correlation between the level of a social network and unmet medical needs. Furthermore, considering the findings of previous studies, which have reported how unmet medical needs are influenced by an array of factors [16], the multidimensional concept of a social network is expected to provide a diversified perspective on the factors influencing unmet medical needs.

In this study, therefore, the characteristics of unmet medical needs in older adults with limited IADL are investigated to examine the effects of the subjects’ social network on their unmet medical needs. Studying how the level of a social network influences unmet medical needs among vulnerable populations will be important for identifying the significance of social networks while establishing measures to secure social support.

## 2. Methods

### 2.1. Study Design

This study was a cross-sectional study that used data from the survey of 2017 National Survey of Older Koreans [17] to identify the factors influencing the unmet medical needs of older adults with limited IADL.

### 2.2. Data and Ethical Considerations

This study analyzed data from the 2017 National Survey of Older Koreans. In accordance with the disclosure and management regulations of the Korean Ministry of Health and Welfare, data were provided for this study, and approval was obtained from the institutional review board of the institution to which the researchers belong (IRB No. SM-202011-074-1). This survey has been conducted every 3 years by the Korean Ministry of Health and Welfare, and the data collection was performed from 12 June to 28 August 2017. The target population of the 2017 National Survey of Older Koreans was older adults aged 65 or over who lived in communities in 17 cities and provinces across the country. A total of 10,299 subjects participated in the survey.

In this study, a total of 2281 subjects who had limited IADL were selected for analysis after excluding those with missing values regarding unmet medical needs (*n* = 114). The ability to perform IADL was measured based on 10 items of the IADL; the subject was considered to have limitations with respect to IADL if assistance from another person was needed for at least one item.

### 2.3. Measurements

#### 2.3.1. Experience of Unmet Medical Needs

For unmet medical needs, the subject was categorized to have experienced unmet medical needs if they answered “yes” to the question, “In the past year, have you not received a medical treatment you thought you needed?” [17,18].

#### 2.3.2. Social Networks

A social network is a multidimensional concept classifiable into two categories: structural characteristics (which indicate quantitative aspects) and functional characteristics (which indicate qualitative aspects) [19]. In this study, the structural characteristics included the size of and contact frequency in a social network (which consists of household type), frequency of contacting close friends, and participation in social activities. The functional characteristics included emotional support, instrumental support, physical support, and financial support.

For structural characteristics, the household type was classified as living as a couple, living alone, or living with others, and the frequency of contacting close friends was measured by how frequently the subject exchanged communications (phone calls, texts, emails, or letters) with friends or neighbors in the past year. In terms of participation in social activities, the subject was considered to have participated if they had attended at least one of the following in the previous year: clubs (club activities), social groups (alumni meetings, etc.), political and social organizations, religious activities, senior citizens community centers, senior citizens’ welfare centers, or volunteer clubs.

For functional characteristics, the support received from the subjects’ children, parents, or spouse was measured. Emotional support was measured by the extent of assistance provided through counseling, while instrumental support was measured by the extent of assistance provided through cleaning, preparing meals, and laundry. Physical support was measured by the extent of assistance provided through physical caregiving and accompaniment to the hospital, while financial support was measured by the extent of assistance provided through regular and nonregular support in cash. Each item was scored based on the Likert scale where 1 was “extremely unlikely”, 2 was “unlikely”, 3 was “likely”, and 4 was “extremely likely”. The subject was given a 0 when they did not have children, parents, or spouses. Higher scores in each category indicated a higher level of support.

#### 2.3.3. Covariate

Sociodemographic characteristics included age, gender, educational level, and annual household income. Educational level was classified into no education, elementary school or less, and middle school or higher. Annual household income was divided into four quartiles from the data of all survey respondents of the 2017 National Survey of Older Koreans, i.e., the first quartile (6.86 million won or less), the second quartile (6.87–9.91 million won), the third quartile (9.92–14.7 million won), or the fourth quartile (14.71 million won or more).

The number of chronic diseases diagnosed by a doctor was considered for the characteristics of the diseases.

### 2.4. Data Analysis

The collected data were analyzed using SPSS Win 22.0 program (SPSS, IBM Corp., Armonk, NY, USA). A descriptive statistics analysis was performed on the measured variables, and the difference in unmet medical needs according to the measured variables was analyzed with X2 tests and *t*-tests. A logistic regression analysis was performed to identify the predictive factors of unmet medical needs according to gender. The level of statistical significance was set at *p* < 0.05.

## 3. Results

### 3.1. Differences in the Experience of Unmet Medical Needs According to the Limited IADL

#### 3.1.1. Characteristics of the Subjects’ Unmet Medical Needs

About 73.0% of the subjects were female in this study, and out of the 2281 total subjects, 361 (15.8%) had experienced unmet medical needs. The difference in the experience of unmet medical needs according to the study’s variables is shown in Table 1. In male subjects, annual household income, frequency of contacting close friends, and participation in social activities had statistically significant differences. In female subjects, educational level, annual household income, number of chronic diseases, household type, frequency of contacting close friends, emotional support, instrumental support, and physical support had statistically significant differences (Table 1).

#### 3.1.2. Differences in the Experience of Unmet Medical Needs According to IADL Items

In male subjects, meal preparation, laundry, walking outdoors, purchasing products, and using public transportation had a statistically significant difference in the experience of unmet medical needs. In female subjects, grooming, financial management, using the telephone, and using public transportation had a statistically significant difference in the experience of unmet medical needs (Table 2).

### 3.2. Effects of a Social Network on Unmet Medical Needs

The factors influencing the experience of unmet medical needs in the subjects were verified through a logistic regression analysis according to gender. In male subjects, annual household income (odds ratio (OR) 3.91, 95% confidence interval (CI) = 1.35–11.31), participation in social activities (OR 2.69, 95% CI = 1.57–4.61), and physical support (OR 0.62, 95% CI = 0.38–0.98) had a statistically significant effect (Table 3).

In female subjects, annual household income (OR 2.35, 95% CI = 1.47–3.77), number of chronic diseases (OR 1.15, 95% CI = 1.08–1.23), living alone in a household (OR 2.04, 95% CI = 1.37–3.04), living with others in a household (OR 1.92, 95% CI = 1.26–2.91), frequency of contacting close friends (OR 0.87, 95% CI = 0.80–0.95), and emotional support (OR 0.70, 95% CI = 0.57–0.86) had statistically significant effects (Table 4).

The Hosmer–Lemeshow goodness of fit test had a *p*-value of 0.05 or greater, thus proving the goodness of fit of the regression model [20]. The Nagelkerke R^2^ value was 0.138 and 0.096 for males and females, respectively.

## 4. Discussion

This study was conducted to identify the effects of a social network on unmet medical needs among older adults with limited IADL who live in a community. Both the structural and the functional characteristics of social networks had a significant effect on the use of medical services, which implies that securing and utilizing a social network is effective for reducing the unmet medical needs of older adults with limited IADL.

Out of the 2281 total subjects, 361 (15.8%) had experienced unmet medical needs. This figure is higher than the findings of a previous study, which reported that 11.6% of the adult subjects had experienced unmet medical needs [21]. The findings of this study correspond to those of another previous study, which reported that the older adult population had a higher incidence of unmet medical needs than the adult population [22]. Considering how 12.6% of the subjects in a study that examined the experience of unmet medical needs in older adults living alone [23] and 9.6% of the subjects in a study conducted among older adults with declined cognitive functions [24] had experienced unmet medical needs, it has been confirmed that the older adult population with limited IADL is experiencing higher incidences of unmet medical needs compared to other vulnerable older adult groups. Therefore, a systematic and comprehensive practical solution needs to be implemented by considering limited functional ability when establishing measures to increase accessibility to medical services for older adults.

When the difference in the experience of unmet medical needs is examined according to the items of IADL, using public transportation was commonly found as a function affecting unmet medical needs in all groups. Specifically, the subjects with limited access to public transportation were more likely to experience unmet medical needs, possibly due to the subsequent limitation on visiting medical facilities to receive treatment. If the causes of unmet medical needs are viewed from the perspectives of availability, accessibility, and acceptability [25], the limitation on using transportation facilities lowers accessibility to medical facilities, thus leading to unmet medical needs [24,26,27]. Since home-visit medical services are not currently provided in South Korea, more attention should be focused on older adults with limited mobility or those using public transportation, while practical solutions should also be devised to reduce their unmet medical needs by providing various services, such as accompaniment to their medical appointments.

The social-network-related factors that affect unmet medical needs varied by gender. The male older adult subjects were less likely to experience unmet medical needs if they had more physical support and were more likely to experience unmet medical needs if they did not participate in social activities. The female older adult subjects were less likely to experience unmet medical needs if they had a higher frequency of contact with close friends and more emotional support, and they were more likely to experience unmet medical needs if they lived alone or lived with a family member other than a spouse. These findings correspond to the findings of previous studies, which reported that individuals experienced unmet medical needs more as a result of fewer frequent contacts and less social support [23,28,29]. With less support from the family and the community, it is more difficult to receive practical support (such as financial support and accompaniment to the hospital for receiving medical services) and obtain necessary information by participating in social activities. Therefore, a social support system needs to be established in addition to providing appropriate information promoting the use of medical services. Volunteer activities for older adults with an efficient network approach applied or various forms of social networks (such as social families) should be established in addition to the vitalization of a support system for the social support system’s efficient operation. In addition, the provision of home-visit medical services could be considered as an appropriate measure. In particular, female older adults are more likely to experience unmet medical needs if they are not coupled, possibly due to financial hardships or the reduction of social networks after bereavement, according to a previous study [18,23]. In South Korea, female older adults who have mostly lived in a patriarchal society are less prepared for their retirement years and are in a more vulnerable position physically, economically, and socially. Therefore, measures should be established to ensure that female older adults form a social network after losing their spouse to encourage their participation in social activities and to build a type of support system for replacing a family.

Meanwhile, financial status was a crucial factor for both men and women. The subjects in the first quartile of annual household income were four times more likely to experience unmet medical needs than those who had a higher income. This finding corresponds with the findings of previous studies [30,31] wherein older adults experienced increased medical expenses and medical needs as they aged, but the medical expense accounted for an increasing portion of their expenses as their income decreased [32]. In particular, the burden of medical expenses is relatively great in households with lower income, which in turn leads to more frequent experiences of unmet medical needs due to the burden of using medical services. Despite the government’s effort to reduce out-of-pocket expenses and support vulnerable social groups’ use of medical services as a part of the health insurance coverage expansion policy, it was discovered in this study that income level was a major factor influencing the experience of unmet medical needs. Thus, sufficient institutional or political complements should be maintained to reduce the gap in accessibility to medical services according to an individual’s ability to pay.

This study has the following limitations: First, the experience of unmet medical needs was evaluated based on a self-reported survey, which may have introduced personal perceptions and subjective judgments. To overcome this drawback, measures for establishing specific political alternatives should be developed and utilized, while the items on particular diseases should be varied in order to measure both subjective and objective unmet medical needs. Second, the changes in the experience of unmet medical needs cannot be examined, since this research is a cross-sectional study that used the 2017 National Survey of Older Koreans. Despite these limitations, this study has significance in that it identified the relationship between unmet medical needs and a social network in older adults with limited IADL, which had not been sufficiently researched using the data from a national survey of older adults. The findings of this study will be utilized as a basis for establishing relevant measures to enable older adults to receive proper medical services by heightening the understanding of the gap between medical service use and the medical needs of older adults with limited IADL.

## 5. Conclusions

This study examined the effects of a social network on unmet medical needs among older adults with limited IADL. The results of this study showed that older adults with limited IADL were highly likely to experience unmet medical needs, which indicates that they are the subjects who will require social investment and relevant solutions. Furthermore, the structural characteristics (frequency of contacting close friends, social activities, and household type) and the functional characteristics (physical and emotional support) of a social network all had effects on the unmet medical needs of older adults with limited IADL. The following proposals can be made based on the findings of this study: First, a support system should be provided continuously to enable older adults to receive medical services based on their social networks. Second, for older adults with limited IADL who live in a community to properly use medical care, more convenient transportation means should be provided in addition to systematic support for accompaniment to the hospital. Moreover, older adults should be provided with training to improve and maintain their financial management skills and their ability to use phones (which are required to use medical services). Third, household type was observed as an influential factor on unmet medical needs among the female older adults in this study, and therefore, qualitative research should be performed to explore in depth the unmet medical needs of female older adults after bereavement.

## Figures and Tables

**Table 1:** Unmet medical needs according to sociodemographic characteristics by gender. *n* = 2281.

Dimension	Variables	Categories (Range)	Unmet Medical Needs					
			Males (*n* = 616)		χ2 or t	Females (*n* = 1665)		χ2 or t
			No (*n* = 538)	Yes (*n* = 80)		No (*n* = 1395)	Yes (*n* = 281)	
			*n* (%) or Mean ± SD			*n* (%) or Mean ± SD		
Covariate	Age (years)		77.65 ± 7.35	78.81 ± 7.09	−1.32	78.47 ± 7.01	78.35 ± 6.61	0.27
	Educational level	No education	127 (23.6)	18 (22.5)	0.05	853 (61.5)	190 (68.3)	7.18 *
		Elementary school	173 (32.2)	26 (32.5)		350 (25.3)	66 (23.7)	
		≥Middle school	238 (44.2)	36 (45.0)		183 (13.2)	22 (7.9)	
	Household income (10,000 won/year)	Q1 (≤686)	178 (33.1)	44 (55.0)	15.63 ***	381 (27.3)	116 (41.6)	24.18 ***
		Q2 (687–991)	149 (27.7)	18 (22.5)		440 (31.5)	81 (29.0)	
		Q3 (992–1470)	130 (24.2)	13 (16.3)		343 (24.6)	52 (18.6)	
		Q4 (≥1471)	80 (14.9)	5 (6.3)		231 (16.6)	30 (10.8)	
	Number of chronic diseases	(0–14)	3.22 ± 2.04	3.36 ± 1.72	−0.61	3.64 ± 1.89	4.17 ± 2.16	−4.16 ***
Structure	Living arrangement	Couple	346 (64.4)	53 (67.9)	0.39	350 (25.2)	46 (16.4)	12.90 **
		Living alone	87 (16.2)	11 (14.1)		538 (38.8)	135 (48.6)	
		Living with others	104 (19.4)	14 (17.9)		499 (36.0)	97 (34.9)	
	Contact with friend and neighbor		1.43 ± 2.20	0.65 ± 1.30	4.51 ***	1.57 ± 2.20	1.03 ± 1.47	5.07 ***
	Social activity	Yes	325 (60.4)	28 (35.4)	17.54 ***	1052 (75.8)	203 (73.0)	1.00
		No	213 (39.6)	51 (64.6)		335 (24.2)	75 (27.0)	
Function	Emotional support		2.69 ± 0.68	2.74 ± 0.64	−0.49	2.82 ± 0.68	2.55 ± 0.78	5.20 ***
	Instrumental support		2.72 ± 0.66	2.72 ± 0.70	0.10	2.53 ± 0.78	2.38 ± 0.78	2.87 **
	Physical support		2.58 ± 0.75	2.50 ± 0.80	0.90	2.53 ± 0.84	2.40 ± 0.87	2.30 *
	Financial support		3.77 ± 0.26	3.79 ± 0.25	−0.63	3.80 ± 0.84	3.77 ± 0.28	1.31

Note. M ± SD, mean ± standard deviation; * *p* < 0.05, ** *p* < 0.01, *** *p* < 0.001.

**Table 2:** Unmet medical needs according to the instrumental activities of daily living (IADL) by gender. *n* = 2281.

Variables	Unmet Medical Needs						
	IADL Limitation	Males (*n* = 616)		χ2	Females (*n* = 1665)		χ2
		No (*n* = 538)	Yes (*n* = 80)		No (*n* = 1395)	Yes (*n* = 281)	
		*n* (%)			*n* (%)		
Grooming	Yes	104 (19.3)	14 (17.7)	0.12	152 (11.0)	48 (17.3)	8.72 **
	No	434 (80.7)	65 (82.3)		1235 (89.0)	230 (82.7)	
Household chores	Yes	377 (70.1)	63 (79.7)	3.15	797 (57.5)	175 (62.9)	2.87
	No	161 (29.9)	16 (20.3)		590 (42.5)	103 (37.1)	
Meal preparation	Yes	364 (67.8)	63 (79.7)	4.63 *	605 (43.7)	133 (47.8)	1.65
	No	173 (32.2)	16 (20.3)		781 (56.3)	145 (52.2)	
Laundry	Yes	328 (61.1)	58 (73.4)	4.48 *	659 (47.5)	149 (53.4)	3.23
	No	209 (38.9)	21 (26.6)		728 (52.5)	130 (46.6)	
Taking medications	Yes	55 (10.2)	12 (15.2)	1.74	94 (6.8)	25 (9.0)	1.71
	No	482 (89.8)	67 (84.8)		1293 (93.2)	253 (91.0)	
Financial management	Yes	148 (27.5)	25 (31.6)	0.58	510 (36.8)	130 (46.6)	9.42 **
	No	390 (72.5)	54 (68.4)		876 (63.2)	149 (53.4)	
Walking outdoors	Yes	90 (16.8)	21 (26.6)	4.50 *	213 (15.4)	54 (19.4)	2.85
	No	447 (83.2)	58 (73.4)		1174 (84.6)	224 (80.6)	
Purchasing products	Yes	85 (15.8)	20 (25.3)	4.38 *	209 (15.1)	54 (19.4)	3.21
	No	452 (84.2)	59 (74.7)		1178 (84.9)	225 (80.6)	
Using the telephone	Yes	211 (39.3)	29 (36.7)	0.19	761 (54.9)	173 (62.2)	5.10 *
	No	326 (60.7)	50 (63.3)		626 (45.1)	105 (37.8)	
Using public transportation	Yes	273 (50.8)	52 (65.8)	6.21 *	765 (55.2)	190 (68.1)	15.91 ***
	No	264 (49.2)	27 (34.2)		622 (44.8)	89 (31.9)	

Note. IADL, instrumental activities of daily living; * *p* < 0.05, ** *p* < 0.01, *** *p* < 0.001.

**Table 3:** Factors associated with unmet medical needs among males.

Dimension	Variables	Categories	B	OR (95% CI)	*p*
Covariate	Age (years)		0.01	1.01 (0.97–1.04)	0.803
	Educational level	No education	−0.25	0.78 (0.40–1.50)	0.454
		Elementary school	−0.01	0.99 (0.56–1.78)	0.983
		≥Middle school		1 (referent)	
	Household income (10,000 won/year)	Q1 (≤686)	1.36	3.91 (1.35–11.31)	0.012
		Q2 (687–991)	0.59	1.79 (0.59–5.43)	0.300
		Q3 (992–1470)	0.15	1.16 (0.37–3.64)	0.794
		Q4 (≥1471)		1 (referent)	
	Number of chronic diseases		0.05	1.05 (0.93–1.19)	0.429
Structure	Living arrangement	Couple		1 (referent)	
		Living alone	0.24	1.27 (0.55–2.92)	0.572
		Living with others	0.24	1.27 (0.63–2.58)	0.503
	Contact with friend and neighbor		−0.17	0.84 (0.71–1.01)	0.063
	Social activity	Yes		1 (referent)	
		No	0.99	2.69 (1.57–4.61)	<0.001
Function	Emotional support		0.30	1.35 (0.84–2.17)	0.211
	Instrumental support		0.08	1.09 (0.64–1.85)	0.766
	Physical support		−0.49	0.62 (0.38–0.98)	0.044
	Financial support		0.72	2.05 (0.72–5.82)	0.180
(Constant)		−6.10			
Correct prediction (%)		87.1			
Hosmer-Lemeshow test		x^2^ = 11.52, df = 8, *p* = 0.174			
Nagelkerke R^2^		0.138			

Note. OR = odds ratio; CI = confidence interval.

**Table 4:** Factors associated with unmet medical needs among females.

Dimension	Variables	Categories	B	OR (95% CI)	*p*
Covariate	Age (years)		−0.02	0.98 (0.96–1.01)	0.246
	Educational level	No education	0.29	1.34 (0.81–2.22)	0.252
		Elementary school	0.21	1.23 (0.72–2.11)	0.444
		≥Middle school		1 (referent)	
	Household income (10,000 won/year)	Q1 (≤686)	0.86	2.35 (1.47–3.77)	<0.001
		Q2 (687–991)	0.26	1.30 (0.81–2.10)	0.276
		Q3 (992–1470)	0.15	1.16 (0.71–1.90)	0.559
		Q4 (≥1471)		1 (referent)	
	Number of chronic diseases		0.14	1.15 (1.08–1.23)	<0.001
Structure	Living arrangement	Couple		1 (referent)	
		Living alone	0.71	2.04 (1.37–3.04)	<0.001
		Living with others	0.65	1.92 (1.26–2.91)	0.002
	Contact with friend and neighbor		−0.14	0.87 (0.80–0.95)	0.003
	Social activity	Yes		1 (referent)	
		No	0.04	1.04 (0.76–1.41)	0.826
Function	Emotional support		−0.36	0.70 (0.57–0.86)	0.001
	Instrumental support		−0.02	0.98 (0.77–1.24)	0.849
	Physical support		−0.03	0.97 (0.78–1.20)	0.780
	Financial support		0.21	1.23 (0.70–2.16)	0.470
(Constant)		−1.55			
Correct prediction (%)		83.3			
Hosmer-Lemeshow test		x^2^ = 10.59, df = 8, *p* = 0.226			
Nagelkerke R^2^		0.096			

Note. OR = odds ratio; CI = confidence interval.

## Data Availability

Data available in a publicly accessible repository.
